# Non-communicable Disease Surveillance in Malaysia: An Overview of Existing Systems and Priorities Going Forward

**DOI:** 10.3389/fpubh.2021.698741

**Published:** 2021-07-06

**Authors:** Arunah Chandran, Shurendar Selva Kumar, Noran Naqiah Hairi, Wah Yun Low, Feisul Idzwan Mustapha

**Affiliations:** ^1^Disease Control Division, Ministry of Health, Putrajaya, Malaysia; ^2^Faculty of Medicine, University of Malaya, Kuala Lumpur, Malaysia; ^3^Asia-Europe Institute, University of Malaya, Kuala Lumpur, Malaysia

**Keywords:** health systems, Malaysia, non-communicable disease, public health, surveillance

## Abstract

In 2012, the World Health Organization (WHO) set a comprehensive set of nine global voluntary targets, including the landmark “25 by 25” mortality reduction target, and 25 indicators. WHO has also highlighted the importance of Non-Communicable Disease (NCD) surveillance as a key action by Member States in addressing NCDs. This study aimed to examine the current national NCD surveillance tools, activities and performance in Malaysia based on the WHO Global Monitoring Framework for NCDs and to highlight gaps and priorities moving forward. A desk review was conducted from August to October in 2020, to examine the current national NCD surveillance activities in Malaysia from multiple sources. Policy and program documents relating to NCD surveillance in Malaysia from 2010 to 2020 were identified and analyzed. The findings of this review are presented according to the three major themes of the Global Monitoring Framework: monitoring of exposure/risk factor, monitoring of outcomes and health system capacity/response. Currently, there is a robust monitoring system for NCD Surveillance in Malaysia for indicators that are monitored by the WHO NCD Global Monitoring Framework, particularly for outcome and exposure monitoring. However, Malaysia still lacks data for the surveillance of the health system indicators of the framework. Although Malaysia has an NCD surveillance in place that is adequate for the WHO NCD Global Monitoring Framework, there are areas that require strengthening. The country must also look beyond these set of indicators in view of the increasing burden and impact of the COVID-19 pandemic. This includes incorporating mental health indicators and leveraging on alternate sources of data relating to behaviors.

## Introduction

The World Health Organization (WHO) has described strengthening surveillance at national level as a priority for tackling Non-Communicable Diseases (NCDs) (1–3). The first global strategy for the prevention and control of NCDs endorsed by the World Health Assembly in 2000 highlighted “surveillance to track and monitor the major risk factors” as a key priority (1). The WHO however has also recognized that several key steps must be taken to strengthen surveillance for NCDs (3):

- NCD surveillance systems should be strengthened and integrated into existing national health information systems;
- All three components of the NCD surveillance framework, i.e., outcomes, exposure/risk factors and health system capacity/response should be established and strengthened;
- Monitoring and surveillance of behavioral and metabolic risk factors in low-resource settings should receive the highest priority; and
- A significant acceleration in financial and technical support is necessary for health information system development in low- and middle-income countries (LMICs).

In 2012, the WHO set a comprehensive set of nine global voluntary targets, including the landmark “25 by 25” mortality reduction target, and 25 indicators (1). These targets, a balance between prevention and treatment, introduced priority and accountability for NCDs in member countries. Malaysia is an Upper-Middle-Income country with a current population of 32.73 million (4). Demographic and behavioral shifts, including rapid urbanization, rapid aging, low health literacy, and an obesogenic environment, have contributed to steady increase in the prevalence of NCDs in the country (5). NCDs accounted for 67% of premature deaths in Malaysia, and over 70% of the burden of disease in 2014 (6).

To strengthen Malaysia's response to the increasing burden of NCDs, the National Strategic Plan for Non-Communicable Diseases (NSP-NCD) 2010-2014 was first published in (7). This was then followed by the National Strategic Plan for Non-Communicable Diseases (NSP-NCD) 2016-2025 published in (8). The NSP-NCD is also aligned to the Government's aspiration for nation building, as stated in the medium-term national plan, i.e., 11th Malaysia Plan 2016-2020 (9). The NSP-NCD 2016-2025 had incorporated the WHO NCD global monitoring targets and underscores the critical importance of an effective surveillance system to track, monitor and interpret trends in risk factors, morbidity, and mortality, as well as responsiveness to policies and public health interventions.

The main objective of this study is to examine the current national NCD surveillance tools, activities and performance in Malaysia based on the Global Monitoring Framework for NCDs and to highlight gaps and priorities moving forward.

## Method

A desk review was conducted from August to October 2020 to examine the current national NCD surveillance activities in Malaysia from multiple sources using several search strategies: literature databases, search engines, targeted websites and consultations with content experts. These strategies were employed for comprehensiveness and to minimize the risk of omitting relevant key information.

The search strategy included the following groups of terms: Non-Communicable Diseases; Surveillance; Policy (guidelines, standards, frameworks); and Malaysia. For this desk review, a search was conducted in August 2020 using PubMed and Google Scholar. A second strategy involved Google Searches for documents published on the Internet. Due to the vast amount of information available on the Internet and for effective time management, authors relied on relevancy ranking within the search engine to ascertain documents most pertinent to the research objectives. Authors also browsed targeted websites of relevant organizations for gray literature. Recommendations for suitable organizations were solicited from content experts on the research team as well as reviewing lists of organizations related to the research topic. Websites of these organizations were searched for potentially relevant documents (reports, guidelines, policy, and program documents). Finally, content experts were contacted to identify other documents for possible inclusion in the review. The authors also reviewed routine data collected by content experts for the monitoring and evaluation of NCD-related activities in Malaysia.

## Results

### Implementation of the NCD Surveillance in Malaysia

Implementation of NCD Surveillance in Malaysia is described in this study based on the three major themes of the WHO Global Monitoring Framework for the 25 indicators: monitoring of outcomes; monitoring of exposure/risk factors; and health system capacity/response. An overview of the findings is shown in Table 1.

**Table 1:** Overview of the 25 indicators for the Global Monitoring Framework for Malaysia.

	**Indicator**	**Name of mechanism & Responsible agency**	**Periodicity**	**Remarks**
**Monitoring of outcomes**				
1.	Premature mortality due to NCDs	National Vital Statistics System (under Department of Statistics Malaysia—DoSM)	Annually	Malaysia does not have a coroner-based system. Almost 50% of deaths are non-medically certified.In October 2017, Malaysia initiated the verbal autopsy (modified WHO methodology) to improve the quality of data on deaths.
2.	Cancer incidence (by type of cancer)	Malaysian National Cancer Registry; under the National Cancer Institute, MOH	Latest report 2012-2016	Issues of under-reporting and delays in reporting
**Monitoring of exposure/risk factors**				
3.	Alcohol per capita consumption	-	-	Data not collected by MOH
4.	Prevalence of heavy episodic drinking	National Health and Morbidity Survey (NHMS) by Institute for Public Health (IPH), MOH	Every 4 years; latest 2019	National population-based survey
5.	Alcohol-related morbidity and mortality	-	-	Data not routinely collected in Malaysia
6.	Physical inactivity (adolescents)	NHMS by IPH, MOH	Every 4 years; latest 2019	National population-based survey
7.	Physical inactivity (adults)	NHMS by IPH, MOH	Every 4 years; latest 2019	National population-based survey; uses the short IPAQ questionnaire
8.	Salt intake	IPH, MOH	2019	Malaysian Community Salt Survey or MyCOSS (2017-2018). Population based, 24-hurinary salt.Non-representative smaller surveys were done in 2012-2015.
9.	Tobacco use (adolescents)	NHMS by IPH, MOH	Every 4 years; latest 2019	National population-based survey
10.	Tobacco use (adults)	NHMS by IPH, MOH	Every 4 years; latest 2019	National population-based survey
11.	Raised blood pressure	NHMS by IPH, MOH	Every 4 years; latest 2019	National population-based survey
12.	Raised blood glucose	NHMS by IPH, MOH	Every 4 years; latest 2019	National population-based survey
13.	Overweight and obesity (adolescents)	NHMS by IPH, MOH	Every 4 years; latest 2019	National population-based survey
14.	Overweight and obesity (adults)	NHMS by IPH, MOH	Every 4 years; latest 2019	National population-based survey
15.	Saturated fat intake	-	-	Data not available in Malaysia
16.	Fruit and vegetable intake	NHMS by IPH, MOH	Every 4 years; latest 2019	National population-based survey
17.	Raised total cholesterol	NHMS by IPH, MOH	Every 4 years; latest 2019	National population-based survey
**Health system capacity/response**				
18.	Drug therapy and counseling	-	-	Survey not done—assume that all eligible patients will receive appropriate treatment based on current related clinical practice guidelines
19.	Essential technologies and medicines	-	-	Survey not done—all healthcare facilities (public and private) will have all essential technologies and medicine available and accessible
20.	Access to palliative care	Palliative Care Services, MOH	-	Data currently not available
21.	Policies to limit saturated fat and eliminate trans-fat	Nutrition Division, MOH	-	Currently Malaysia do not have any explicit policies on this
22.	HPV vaccination	Family Health Development Division, MOH	Annual	Data collection through an electronic monitoring mechanism (MOH school health teams)
23.	Marketing to children	Nutrition Division, MOH	-	Currently, it is self-regulated – selected companies and selected food
24.	Hep. B vaccination	Family Health Development Division, MOH	Annual	Data collection through an electronic monitoring mechanism (mostly MOH healthcare facilities)
25.	Cervical cancer screening	Family Health Development Division, MOH	Annual	Data collection through an electronic monitoring mechanism (mostly MOH healthcare facilities)

### Monitoring of Outcomes

For the data on premature mortality due to NCDs, Malaysia relies on the national vital statistics data by the Department of Statistics, Malaysia (DoSM) to monitor the unconditional probability of dying between ages of 30 and 70 from cardiovascular diseases, cancer, diabetes, or chronic respiratory diseases. However, due to the absence of a coroner-based system, almost 50% of all deaths in the country are non-medically certified.

In order to improve the quality of causes of death data collected by DoSM, the government has introduced the Verbal Autopsy (VA) initiative to improve the quality of data for the death registration (10, 11). This is achieved by increasing the proportion of deaths with medically certified causes through the VA initiative. This was initiated in 2017, through a national circular by the Director General of Health Malaysia, mandating a national system for verification of non-medically certified deaths using the VA methodology (12). The circular prescribes that causes of death for non-medically certified deaths should be verified using VA questionnaires. A mechanism was also established to facilitate data sharing and collaboration between local offices of the National Registration Department (NRD), DoSM, and MOH.

To monitor the incidence of cancer in Malaysia, the Malaysian National Cancer Registry (MNCR) was established in all states in 2007 under the Public Health Program of the MOH (13). The MNCR receives notifications from all data source providers in government and private health facilities using a standardized format. Comprehensive cancer registration is achieved through data obtained from a combination of sources using both passive and active case finding methods, (a) notifications by the medical profession; (b) pathology records; (c) hospital discharges records; (d) mortality data from the NRD and hospital death notification; and (e) Hospital based cancer registry. For cancer cases obtained from sources other than physician notifications, the data will be checked against known registered cases in the registry (14).

In Malaysia, cancer is not a notifiable disease. However, in April 2020, the Director General of Health Malaysia issued an administrative circular, mandating reporting of all cancer patients to MNCR by all registered medical practitioners in both public and private healthcare facilities involved in diagnosing and treating cancer. Public or private health facilities that are not involved directly in the confirmation of a cancer diagnosis, but are involved in cancer treatment, are also encouraged to notify to MNCR if they have complete diagnosis information (for cancer cases that have never been notified).

### Monitoring of Exposure/Risk Factors

The National Health and Morbidity Survey (NHMS) for NCD risk factors, which is based on the WHO STEPs survey (15), is a population based cross-sectional survey administered with an interval of four years to monitor NCD risk factors among the adult population (16). There are also other nationally representative surveys that monitor adolescents, and the elderly. This survey monitors all the NCD risk factors monitored in the Global NCD Framework, except for salt and saturated fat intake. A separate population-based survey to monitor salt intake called the MyCOSS study which was specially commissioned in 2018 (17). Saturated fat intake is not routinely monitored at the population level in Malaysia, although data on the population-level overall fat intake is available from NHMS (18).

NHMS uses a standard methodology for a household survey for adults and a school-based survey for adolescents. A multi-stage stratified sampling method is used to produce a nationally representative data. It covers both urban and rural areas in all states in Malaysia. Data is collected by face-to-face interview using structured questionnaires, as well as self-administered questionnaires. Clinical assessment and biochemistry tests are also conducted. Since 2015, the survey information was collected electronically using handheld devices.

### Health System Capacity/Response

MOH does not routinely conduct healthcare facilities surveys to enable reporting for the indicators “drug therapy and counseling” and “essential technologies and medicines.” All healthcare facilities in Malaysia are equipped with essential technologies and first-line medications, both available and accessible, to the population, as Malaysia provides universal health coverage (UHC) to its population (19, 20).

Coverage of the hepatitis B immunization from MOH healthcare facilities is monitored using the HIMS e-reporting system. It is a web-based reporting system, through which, selected health related data from MOH healthcare facilities are transacted to ensure timely and quality health information. However, for non-MOH healthcare facilities, aggregated data is collected through a manual paper-based system.

The HPV Immunization Program in Malaysia was launched in August 2010 and added to the list of the National Immunization Program (NIP), which provides selected vaccines free of charge to all residents as a public health service (21, 22). High rates of school enrolment for 13-year-old (96.0%) and retention of female students in secondary schools have made it possible for the HPV vaccination to be integrated into the School Health Service Program (under MOH) and ensures equal access to the HPV vaccine between urban and rural area (22). The immunization coverage is monitored through a dedicated web-based reporting system.

Cervical cancer screening is conducted in Malaysia as an opportunistic screening. Data on coverage of screening in MOH healthcare facilities is collected via the HIMS e-reporting system. For some non-MOH healthcare facilities, aggregated data is collected through a manual-based system. The NHMS 2011 and 2019 also collected and reported data on the prevalence of pap-smear screening among eligible populations.

Malaysia does not routinely collect data on access to palliative care, which is assessed by morphine-equivalent consumption of strong opioid analgesics (excluding methadone) per death from cancer. MOH launched the “National Palliative Care Policy and Strategic Plan 2019-2030” in (23) in order to strengthen overall policies related to palliative care, including surveillance.

The MOH gazetted the mandatory declaration on the amount of trans fatty acids together with the amount of saturated fatty acids, monounsaturated fatty acids and polyunsaturated fatty acids on nutrition labels, when a claim is made regarding the amount and/or types of fatty acids in (24). In addition, the Malaysian Food Act 1983 Food (Amendment) Regulation 2003 has also made provisions for nutrient content claims on low and free trans fatty acids in foods. However, there is no law or policy as yet to limit the amount of trans fat in food. MOH also does not have specific policies related to marketing of foods and non-alcoholic beverages high in saturated fats, trans fatty acids, free sugars, or salt.

## Discussions

### Gaps and Challenges for NCD Surveillance in Malaysia

Currently, there is a robust monitoring system for NCD Surveillance in Malaysia for indicators that are monitored by the WHO NCD Global Monitoring Framework, particularly for outcome and exposure monitoring. However, Malaysia still lacks data for the surveillance of the health system indicators of the framework.

For monitoring of mortalities, despite the existence of laws on civil registration in Malaysia that requires registration of all deaths occurring in the country, deaths that occur outside a health facility can still be certified by a non-medical enforcement officer. In 2018, this was approximately half the death that occurred. For these deaths, the top causes cited are often non-medical terms such as “old age” (10). The coroner system for death inquiry only applies for investigation of suspicious and violent deaths, and other deaths as specified under the Criminal Procedure Code [Act 593]. Due to the socio-cultural context of the need for rapid burial of the dead in Malaysia, it is unlikely that any changes to the registration of deaths system will occur soon.

With regards to cancer incidence, there are possibilities of under-reporting of cancer cases in the country, due to the lack of mandatory requirement for reporting by medical practitioners in the country. We also postulate that some patients with cancers may not seek treatment at healthcare facilities and instead seek alternative non-proven therapies elsewhere. There are several studies that have shown delays in presentation and diagnosis of cancer patients in Malaysia due to these factors (25–27).

The challenges to fulfill the criteria for indicators in the health system category arises from the dichotomous healthcare system in Malaysia, whereby the public and private healthcare services co-exist and there is an absence of a national and interconnected electronic medical record (EMR) system. There is also an absence of a regular facility-based surveillance for NCD management at both primary and secondary care levels.

Although there are already several web-based systems in place to collect data for hepatitis B vaccination, HPV vaccination and cervical cancer screening, these still require primary data entry by the healthcare providers. Limited availability of computers, poor connectivity, limited time for data entry by healthcare providers may affect quality. While data submission for public facilities can be made compulsory by a top-down administrative circulars, there is no regulatory mechanism for mandatory data submission by private healthcare facilities. However, some limited data on private healthcare facilities are available through *ad hoc* primary care and hospital surveys (28, 29).

### Challenges in NCD Surveillance Due to COVID-19

There are two major challenges posed by COVID-19 and the movement restrictions on NCD surveillance, firstly on data collection, and secondly monitoring for the NCD-related consequences due to COVID-19 and the movement restrictions. A recent review has shown people living with NCDs are at higher risk of more severe infection and mortality due to COVID-19, and that the movement restrictions have already caused disruptions to the screening, treatment, self-care and surveillance of NCD patients (30).

Supplementation of mortality data with VA has been affected due to the COVID-19 pandemic. Implementation of the VA initiative requires home visits and face-to-face interviews. During the movement restrictions, members of the public are also reluctant to come into contact with healthcare workers. In addition, the healthcare workers assigned for VA work have been re-assigned to support COVID-19 response. The re- assignment of healthcare workers has also disrupted data collection and data entry at primary healthcare facilities.

For population-based surveys, despite the fact that data collection can still be continued under the new COVID-19 standard operating procedures (SOPs), modifications have to be made in terms of the work processes to limit face-to-face interactions. For example, for NHMS 2020, blood taking was conducted at the study locations. These modifications, that also includes the use of PPE by the survey enumerators, add to the costs of data collection. This added to the fear of COVID-19 infection risk by the general population would negatively impact the response rate of participants and the quality of data collected.

We would need to look beyond the NCD WHO Global Monitoring Framework as the current NCD surveillance systems in many LMICs, including Malaysia, will not be able to monitor the NCD-related consequences due to COVID-19 in a timely manner. For Malaysia, firstly, this will require strengthening surveillance of mental health problems in the country, focusing on vulnerable groups. Some data is currently available on the utilization of mental health and psychosocial support services helplines that have been made available during this COVID-19 pandemic, as well as other existing social support services by the government. However, the coverage, representativeness and utility of this data is limited.

Secondly, the disruption to the availability and accessibility of primary healthcare services, from both the healthcare providers' perspective as well as the patients' perspective, must also be monitored at regular intervals. There is currently no existing efficient mechanism for such monitoring in the country. Thirdly, clinical process indicators and several selected clinical outcome indicators must also be measured at regular intervals. In the absence of electronic medical records, effective collection of data for these variables will require novel approaches.

### Moving Forward

The adoption of electronic health records (EHR) could improve the availability and accuracy of data from healthcare facilities. Databases of healthcare facilities providing longitudinal electronic health records data allow patterns to be detected in real time, as well as rapid shifts in morbidity, risk factors and delivery of health care. These databases become a reliable repository for NCD surveillance if it can be cross-referenced across public and private health facilities and provide a platform for meaningful local action.

In Malaysia, data capture procedures are mostly done manually and conducted retrospectively by tracing the patient's health characteristics and care characteristics. This approach is costly with a high risk of missed values and imprecisions. Research has shown that the use of EHRs has the potential to help enhance the privacy, efficiency and cost- effectiveness of healthcare services (31). However, many factors may lead to the low acceptance of the EHR method, including high costs, respect for the data of the privacy user, social effects, too difficult to use method and lack of training (32, 33).

Under current circumstances, such a comprehensive EHR will be beyond the country's financial capacity. Therefore, one option moving forward could be an augmented sentinel surveillance at the primary care level can provide important information on the progression of the different aspects of NCD services and utilization on a routine basis (34).

Beyond population-based surveys, we should also leverage on the use of existing technologies to closely monitor behavioral NCD risk factors. This will enable us to monitor both the current status as well as the immediate impact of any intervention activities or initiatives relating NCD-related behaviors.

In Malaysia, an estimated 87.4% of the population are internet users (35). Smartphones remained at the most common device used to access the Internet with 93.1% of users use smartphones to go online (35). Online technologies such as smartphone apps, messaging platforms and e-learning are changing how improving health education programmes are delivered. The WHO's “Be He@lthy, Be Mobile” Initiative for example, has created a population-wide digital health preventive toolkit, including SMS notifications, mobile physical application, and digital health education (36).

In one study, phone usage data provided behavioral indicators that were strongly related to depressive symptom severity (37), while another international study of smartphone data highlighted global physical activity inequalities and potential areas for intervention (38). Another study found a linkage between anger and fatigue expressed on Twitter, to an increased risk of heart disease (39). Social media has also shown the association between likelihood of alcohol consumption and number of Facebook likes (40).

Additionally, high-frequency and high-resolution sensor data, from wearable technologies to environmental sensors offer the prospect of identifying new NCD risk factors, insights into lifestyle behaviors, and tracking patient engagement in self-care and chronic illnesses management (41, 42). For example, such sensors have been extensively used for remote monitoring of patients with cardiovascular disease (43).

Mining of virtual digital trails such as social media data offers the opportunity to assess self- reported health related attitudes and behaviors pertaining to NCDs and their risk factors in near real-time and can complement other NCD surveillance data (44, 45). Real-life digital trails are signals produced by people's everyday actions, recorded digitally through devices and sensors measuring individuals' movements and behaviors. There is need to innovate and expand NCD Surveillance with the use of big data and artificial intelligence (AI). The practicality of these solutions remains to be seen but it nevertheless remains an interesting option. Achieving NCD Surveillance using big data and AI needs to consider legal, ethical and technical challenges (46).

## Conclusion

NCD surveillance is an essential component in a country's response on the increasing burden of NCDs, affecting the health outcomes and quality of life of the population, as well as the negative impact on a nation's economic development.

Although the current NCD surveillance in Malaysia is able to provide most of the data for the indicators in the WHO NCD Global Monitoring Framework, there is a need for the country to look beyond these set of indicators. There is also an urgent need for strengthening the surveillance, incorporate the appropriate mental health component indicators, as well leverage on alternate sources of data (e.g., social media data), in view of the expected increasing burden of NCDs and current challenges in planning, monitoring and evaluation of interventions.

While much attention has been put on the burden of chronic physical illness, mental health is an area that tends to be neglected. It is hoped that the whole-of-government approach can be strengthened with the involvement of partnership with various stakeholders to prevent and control NCDs in Malaysia. The current COVID-19 pandemic and movement restrictions, resulting in NCD-related collateral damages, will provide further justification for the need for strengthening the NCD surveillance systems in all countries, including Malaysia.

## Author Contributions

AC and SS conceived, designed the framework, and prepared the initial draft. AC, SS, and FM further refined the draft based on interpretation of the data. All authors reviewed and approved the final manuscript.

## Conflict of Interest

The authors declare that the research was conducted in the absence of any commercial or financial relationships that could be construed as a potential conflict of interest.
